# Drinking Water Turbidity and Emergency Department Visits for Gastrointestinal Illness in New York City, 2002-2009

**DOI:** 10.1371/journal.pone.0125071

**Published:** 2015-04-28

**Authors:** Jennifer L. Hsieh, Trang Quyen Nguyen, Thomas Matte, Kazuhiko Ito

**Affiliations:** 1 Bureau of Communicable Diseases, New York City Department of Health and Mental Hygiene, New York, New York, United States of America; 2 Applied Research, Community Health Epidemiology, and Surveillance Branch, Population Health Division, San Francisco Department of Health, San Francisco, California, United States of America; 3 Bureau of Environmental Surveillance and Policy, New York City Department of Health and Mental Hygiene, New York, New York, United States of America; Centers for Disease Control, TAIWAN

## Abstract

**Background:**

Studies have examined whether there is a relationship between drinking water turbidity and gastrointestinal (GI) illness indicators, and results have varied possibly due to differences in methods and study settings.

**Objectives:**

As part of a water security improvement project we conducted a retrospective analysis of the relationship between drinking water turbidity and GI illness in New York City (NYC) based on emergency department chief complaint syndromic data that are available in near-real-time.

**Methods:**

We used a Poisson time-series model to estimate the relationship of turbidity measured at distribution system and source water sites to diarrhea emergency department (ED) visits in NYC during 2002-2009. The analysis assessed age groups and was stratified by season and adjusted for sub-seasonal temporal trends, year-to-year variation, ambient temperature, day-of-week, and holidays.

**Results:**

Seasonal variation unrelated to turbidity dominated (~90% deviance) the variation of daily diarrhea ED visits, with an additional 0.4% deviance explained with turbidity. Small yet significant multi-day lagged associations were found between NYC turbidity and diarrhea ED visits in the spring only, with approximately 5% excess risk per inter-quartile-range of NYC turbidity peaking at a 6 day lag. This association was strongest among those aged 0-4 years and was explained by the variation in source water turbidity.

**Conclusions:**

Integrated analysis of turbidity and syndromic surveillance data, as part of overall drinking water surveillance, may be useful for enhanced situational awareness of possible risk factors that can contribute to GI illness. Elucidating the causes of turbidity-GI illness associations including seasonal and regional variations would be necessary to further inform surveillance needs.

## Introduction

Many studies have examined the relationship between turbidity as an indicator of drinking water quality and measures of endemic gastrointestinal (GI) illness. The methods, quality, and locations of the studies have varied, and a 2007 review showed mixed results even for studies meeting standardized quality criteria [1]. Differences in analytical methods, regional water quality, drinking water exposure, and case definitions for GI illness among other factors, may influence these results. Analyses of turbidity and healthcare visits for GI illness conducted in drinking water systems in Philadelphia, Atlanta, and Vancouver have shown small positive associations between turbidity and endemic GI illness [2–5]. Another conducted in Edmonton, CA found no association [6].

Turbidity is a standard drinking water quality indicator which is related to the amount and physical characteristics of suspended particles but does not indicate the type or source of particles. It is quickly and easily measured at low cost and can be a useful early indicator of water quality changes. Turbidity can be associated with increased runoff entering a system and microbial loading, as can occur following a precipitation event [7, 8] and particles contributing to turbidity can reduce the efficacy of chlorine in inactivating microbes [9]. The components contributing to turbidity can vary between watersheds, seasons, and years. Turbidity can increase related to changes in source water such as precipitation events or wind-driven mixing and also changes that can occur within the drinking water distribution system such as low pressure events. Increases in turbidity do not necessarily indicate a health risk as different sources and particle types contribute to turbidity and not all turbidity increases are associated with contamination.

Increased turbidity has been associated with previous waterborne outbreaks including the *Cryptosporidium* outbreak in Milwaukee, Wisconsin and an *E*. *coli* outbreak in Walkerton, Ontario [10, 11] and increased turbidity was associated with emergency department (ED) visits for GI illness even before the large outbreak in Milwaukee [12]. Such water-borne outbreaks are rare, but evaluating whether there is an association of increased turbidity with GI illness may help enhance early detection of water quality issues.

This study was conducted as part of a larger project focused on enhancing systems for the rapid detection of water contamination events using currently collected water quality and health data. Available water quality and health outcome data were reviewed for potential utility in a rapid detection system. Turbidity and ED GI illness visits were selected based on data availability, quality, timeliness, and support in the literature for use in this type of surveillance. ED data for patients presenting with a clinical syndrome consistent with GI illness (“syndromic data”) is more rapidly collected and reported to the New York City Department of Health and Mental Hygiene (NYC DOHMH) than traditional surveillance data such as positive clinical laboratory test results. ED syndromic data has been used to aid traditional surveillance. In 2003, ED syndromic analysis helped rapidly identify an increase in GI illness that was not detected by laboratory surveillance following a citywide power outage [13]. Interpretation of this signal was aided by supporting information and this analysis highlighted the utility of syndromic analysis in the context of overall public health surveillance.

There have been no known outbreaks of GI illness related to New York City’s (NYC) current drinking water system. Nevertheless, surface water systems can be vulnerable to increased turbidity during extreme rain events, disturbances to the distribution system such as low pressure events and potentially to accidental or intentional contamination [7, 14, 15]. NYC has put in place a variety of systems for detection of such incidents including monitoring of water system and water quality parameters and certain public health parameters including ED GI illness. For this study, we conducted a retrospective time-series analysis of the relationship between drinking water turbidity and ED diarrhea visits in NYC during 2002–2009, to improve our understanding of how monitoring these data streams together may help enhance our systems for rapid detection of some types of water contamination events.

## Materials and Methods

### Exposure Data

The NYC drinking water system is an unfiltered drinking water system which receives surface water from 3 watersheds located North of NYC. This system includes 19 lakes and controlled reservoirs; water volume from reservoir systems varies daily and is actively managed based on quality and availability. Daily turbidity measurements for January 1, 2002 to December 31, 2009 provided by the NYC Department of Environmental Protection (NYC DEP) included data from over 375 sites within the NYC distribution system (~ 40 sampled/day) and 3 key point sites, one from each reservoir system: the Catskill, Delaware, and Croton[16]. As a daily measure of NYC distribution system turbidity, we computed the daily median turbidity among NYC distribution system sites sampled to limit the influence of localized extreme values (“NYC turbidity”). For source water turbidity we computed the daily flow-weighted average from the source water sites (“source water turbidity”). In cases where there were missing values in source water turbidity data, we imputed values by replacing missing values with the monthly average value or yearly average value if monthly average was not available. Summary statistics of daily turbidity in NYC and source water are shown in the supporting information S1 Table. One outlier (4.6 ntu) that occurred in January 2006 for the source water turbidity was removed from further analysis because a review of historical records indicated that it was associated with an interruption of normal systems operations and due to subsequent changes of system configurations that day it was not representative of water entering the distribution system (and was not reflected in the New York City distribution turbidity samples that day).

### Outcome Data

NYC DOHMH receives daily ED visit records from NYC hospitals. These visits are categorized by syndrome type by scanning for key words in the chief complaint for each visit [17]. Keyword search for the ED diarrhea syndrome includes mention of diarrhea, enteritis, gastroenteritis, loose stools, and stomach flu. Daily counts of diarrhea syndrome ED visits were used as the outcome variable in this study. From 2002 to 2004, the number of hospitals contributing data varied, but, for the remaining study period, daily data for approximately 95% of all ED visits in NYC were available.

### Weather covariates

The 24-hour average temperature and 24-hour cumulative precipitation data for LaGuardia airport were obtained from the National Oceanic and Atmospheric Administration, National Climatic Data Center (2009) Global Summary of the Day database.

### Exploratory Analysis

We first conducted exploratory data analyses to characterize temporal patterns of turbidity, diarrhea ED visits, and potential confounders such as weather, to inform regression models. To characterize relative temporal variance contributions from seasonal trends, day-of-week, and random components, we conducted spectral analyses [18–20] of turbidity and diarrhea ED visits. We used modified Daniel smoothers to compute smooth season-specific periodograms, applying several spans of smoothing over frequency intervals [21], and then pooling them across years by frequency [18].

To characterize bivariate short-term temporal relationships among weather, turbidity, and diarrhea ED visits, we first removed the influence of shared trends, seasonal cycles, and day of week patterns from the short-term relationships among the variables by de-trending each time series in a generalized linear model using natural cubic splines with six degrees of freedom per season and a day-of-week indicator variable.

To assess bivariate temporal relations among variables that may change across seasons, a cross-correlation function (CCF) was then computed using pairwise complete observations between the residual time series with multiple time lags (-10 to +10 days) at each of the twelve months using the multiple years’ data for the three months surrounding that month (e.g., for February CCF, the de-trended data for January, February, and March for multiple years were used). This method allowed for assessment of the changing pattern of associations, if any, between two variables across seasons with sufficient statistical power (i.e., r ~ 0.1 would be significant with six years of data in a 3-month block).

### Regression Models

Percent excess risk (% ER) of daily diarrhea ED visits for turbidity was estimated using a quasi-liklihood Poisson time-series regression model, adjusting for temporal trends and seasonal cycles, immediate and delayed temperature effects, day of week, and holidays. The analysis was stratified by season, based on the seasonal variation in turbidity, ED outcomes and CCFs. Thus, the number of observations considered was 736 days for spring and summer, 728 days for fall, and 722 days for winter. The extent of lagged days considered for water quality and meteorological variables was based on exploratory analysis of these data described above. The main regression model had the following model specification for each season:

Log[E(Yt)]=α+βt-i*turbidityt-i+ns(studyday,df=8/seasonperyear)+ns(tempt,df=3)+ns(avg.tempt-1:3,df=3)+factor(day-of-week)+holiday_indicatort-0:1

where Y_t_ is the number of diarrhea ED visits on day t; α is an intercept; β _t-i_ is a regression coefficient for the turbidity on the lag i day; ns indicates a smooth function of a predictor using natural cubic splines; temp_t_ is ambient temperature on the same day; avg.temp_t-1:3_ is the average of past 1 through 3 day lagged temperature; day-of-week is a class variable; and holiday_indicator_t-0:1_ indicates the day of or before a federal holiday. The quasi-likelihood Poisson model adjusts the standard error of the regression coefficients to accommodate possible Poisson over-dispersion. We used natural cubic splines of study days to adjust for potentially confounding temporal trends (e.g., the influence of norovirus) and sub-seasonal cycles. Since the study days here are sequence of numbers for the entire study period, this smooth term also adjusts for the long-term trends. In choosing the degrees of freedom for this temporal adjustment, we evaluated the statistical significance of the first-order autocorrelation of the residuals from the models using 2 to 14 degrees of freedom, in 2 degrees of freedom increment, per season per year. Based on this evaluation, using 8 degrees of freedom per season for each year for spring, summer, and fall, was sufficient 12 degrees per season per year for winter was required.

To adjust for temperature effects, we also included in our base model natural cubic splines of the same-day and the average of past 1- through 3-day lagged temperature with 3 degrees of freedom over the range for each term. The choice of these lags was based on the CCF results (not presented). NYC precipitation was not associated with diarrhea ED visits and was not included in the regression model. Risks were estimated at lags 0 through up to 10 days for inter-quartile range (IQR) increases of turbidity for spring, summer, fall and winter seasons.

For the main model described above, the model diagnostics were conducted by evaluating the models’ Pearson residuals in several ways: (1) examining time-series plots; (2) examining the residuals vs. fitted values; (3) examining the autocorrelation function (ACF) and partial autocorrelation function (PACF) of the residuals; and, (4) examining ACF and PACF of the square of the residuals.

We conducted several additional analyses to examine the sensitivity of the risk estimates to alternative model specifications and to check the consistency of our main findings with the causal inference by examining the influence of different subsets of exposure and outcome data. Thus, we: (1) examined sensitivity of risk estimates by using a negative binomial model, which is also used for over-dispersed count data (Gardner et al., 1995) and compared both the regression coefficients and standard errors obtained from the quasi-likelihood Poisson models; (2) assessed effect of removal of the highest NYC turbidity values; (3) checked the sensitivity of risk estimates to alternative degrees of freedom to adjust for within-season variation in ED visits (6 through10 degrees of freedom per season were examined); (4) fit a distributed lag model to estimate the impact of multi-day associations over lag 0 through 13 days through evaluating 2^nd^, 3^rd^, 4^th^-order polynomial shapes using an R package ‘dlnm’ (distributed lag non-linear model; [22]); (5) examined sensitivity of risk estimates to four-year moving seasonal blocks of seasonal data subsets; and (6) examined sensitivity of risk estimates to alternative temperature model specifications.

All statistical analyses were conducted using the R statistical software package (version 2.15.3: R Development Core Team 2013).

## Results

**Fig 1:**
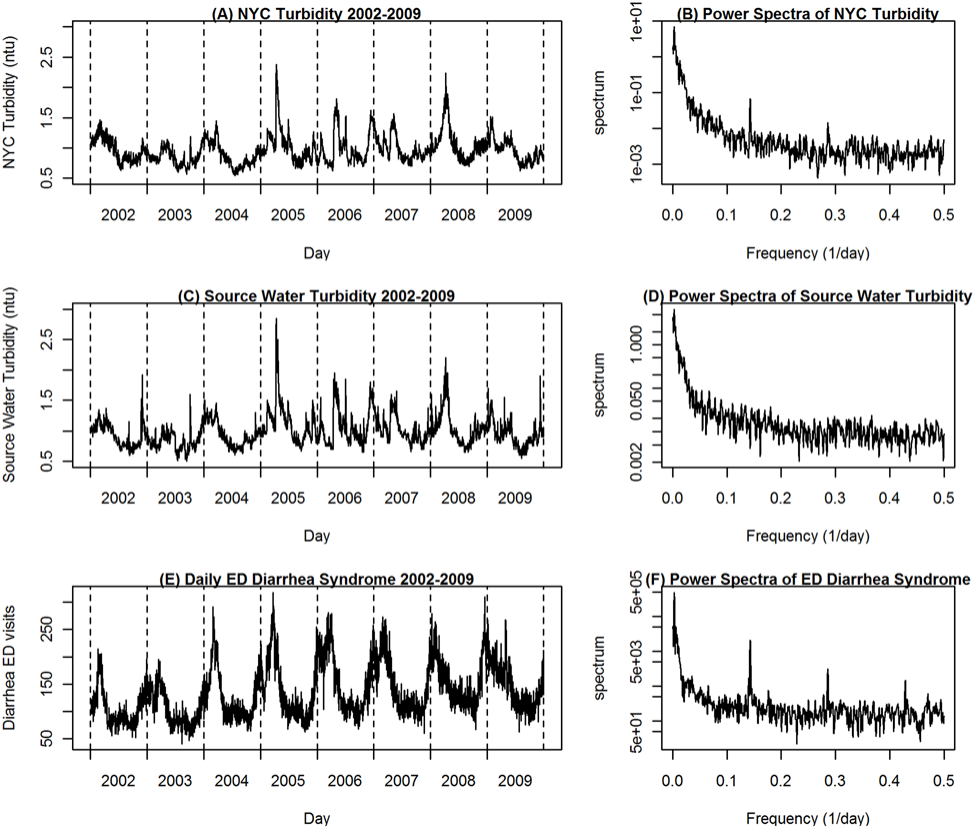
Time series and spectral analysis plots for NYC turbidity, source water turbidity, and diarrhea ED visits from 2002–2009.

The median daily NYC turbidity values were highest in spring, followed by winter (Fig 1) and no daily median NYC turbidity data was missing. Over the study period, an average of 96% of NYC source water by volume came from the Catskill and Delaware watersheds and 4% from the Croton watershed (S1 Table). Less than 2% of Catskill and Delaware system and 19.8% of Croton reservoir system turbidity values were imputed due to missing data and only a single daily value of source water turbidity was excluded as an outlier (4.6 ntu). Croton data had a very small influence on source water flow-weighted average due to the limited contribution by volume from this system. The correlation between source water turbidity from all sites and turbidity from just Catskill/Delaware sites was 0.995. NYC turbidity and source water turbidity showed a similar seasonal fluctuation that typically peaked in the spring and a similar pattern of varied timing and intensity from year to year (Fig 1). Time-series depiction of diarrhea ED visits showed strong seasonal patterns with yearly peaks in the winter and a moderate upward trend over the study period due to an increasing number of EDs included in syndromic data over time and increasing overall utilization at EDs beginning in December of 2004. Multiple hospitals from each borough of NYC contributed to the syndromic system each year for the entire study period. The 0–4 age group accounted for 37.3% of all diarrhea ED visits, 12.1% of all ED visits, and 7% of the population. Multiple hospitals from all boroughs contributed data to the syndromic system for each year of this study.

**Fig 2:**
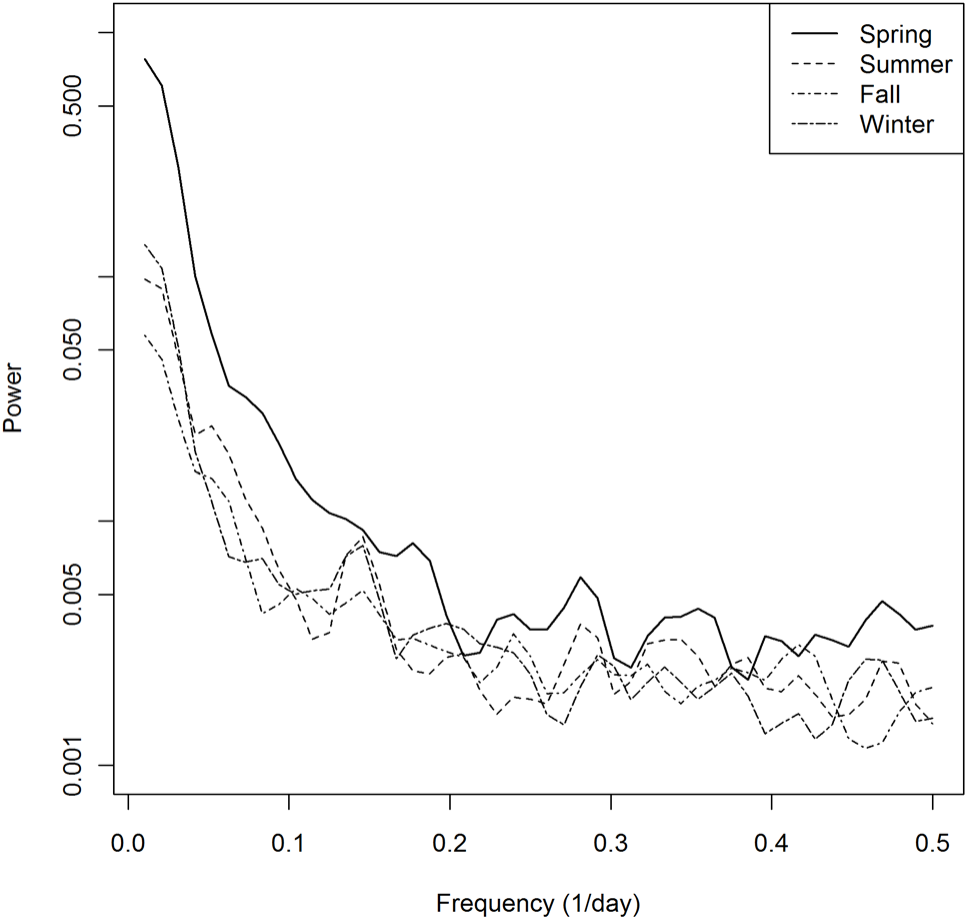
Season specific power spectra of median NYC turbidity, 2002–2009.

The power spectra for NYC turbidity (Fig 1) showed a major variance contribution in the frequency range corresponding to seasonal cycles. A day-of-week pattern was apparent in the frequency range corresponding to day-of-week cycle frequencies (0.14/day or 7-day cycle and its harmonics) for both NYC turbidity and diarrhea ED but not for source water turbidity. The season-specific spectra (Fig 2) show that the variance contributions for the spring period are larger than those for other seasons in nearly all the frequency ranges.

**Fig 3:**
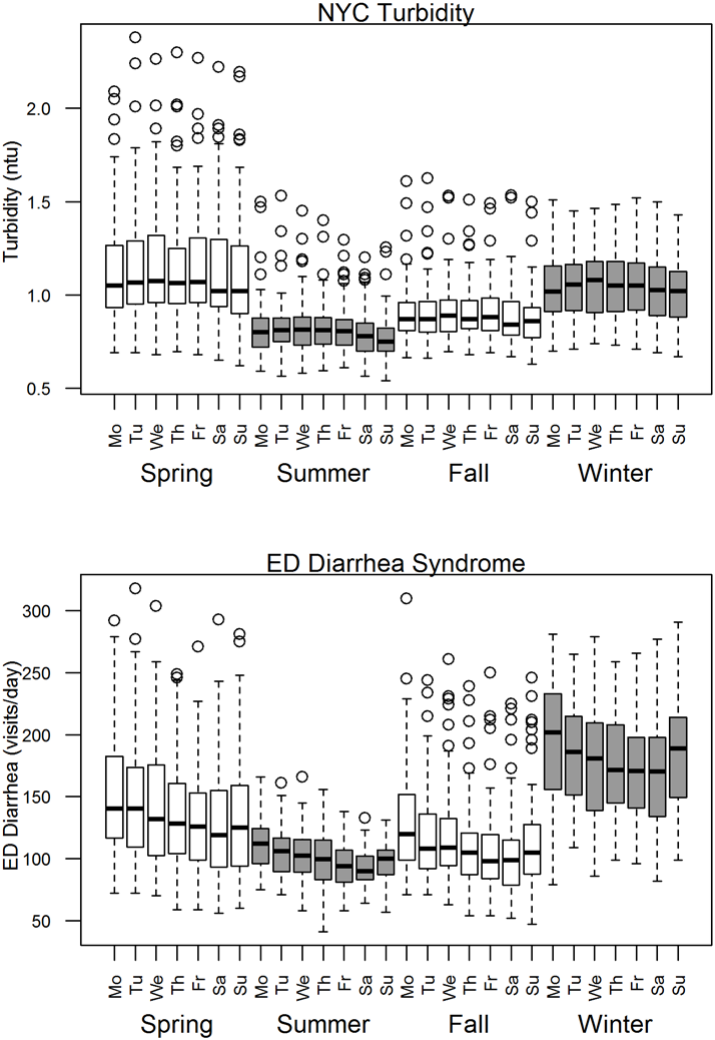
Box plot of day-of-week patterns for median NYC turbidity and diarrhea ED visits by season, 2002–2009.

While the median values of NYC turbidity were similar in spring and winter, variability was much greater in spring than in winter (Fig 3). The diarrhea ED visits series also exhibited a day-of-week pattern but a somewhat different seasonal pattern with higher values in the winter than spring and lowest in summer, further indicating the need for season-specific analyses.

To guard against over-interpretation of chance findings, we focused on identifying consistent patterns (e.g., associations at consecutive lags) rather than statistical significance of associations at individual lagged days. There was no association between diarrhea ED visits and precipitation for all ages or for any age group individually (not shown). Diarrhea ED visits showed positive associations with ambient temperature on the same day during the cold season and early spring (not shown), suggesting the need to include temperature in models as a potential confounder.

**Fig 4:**
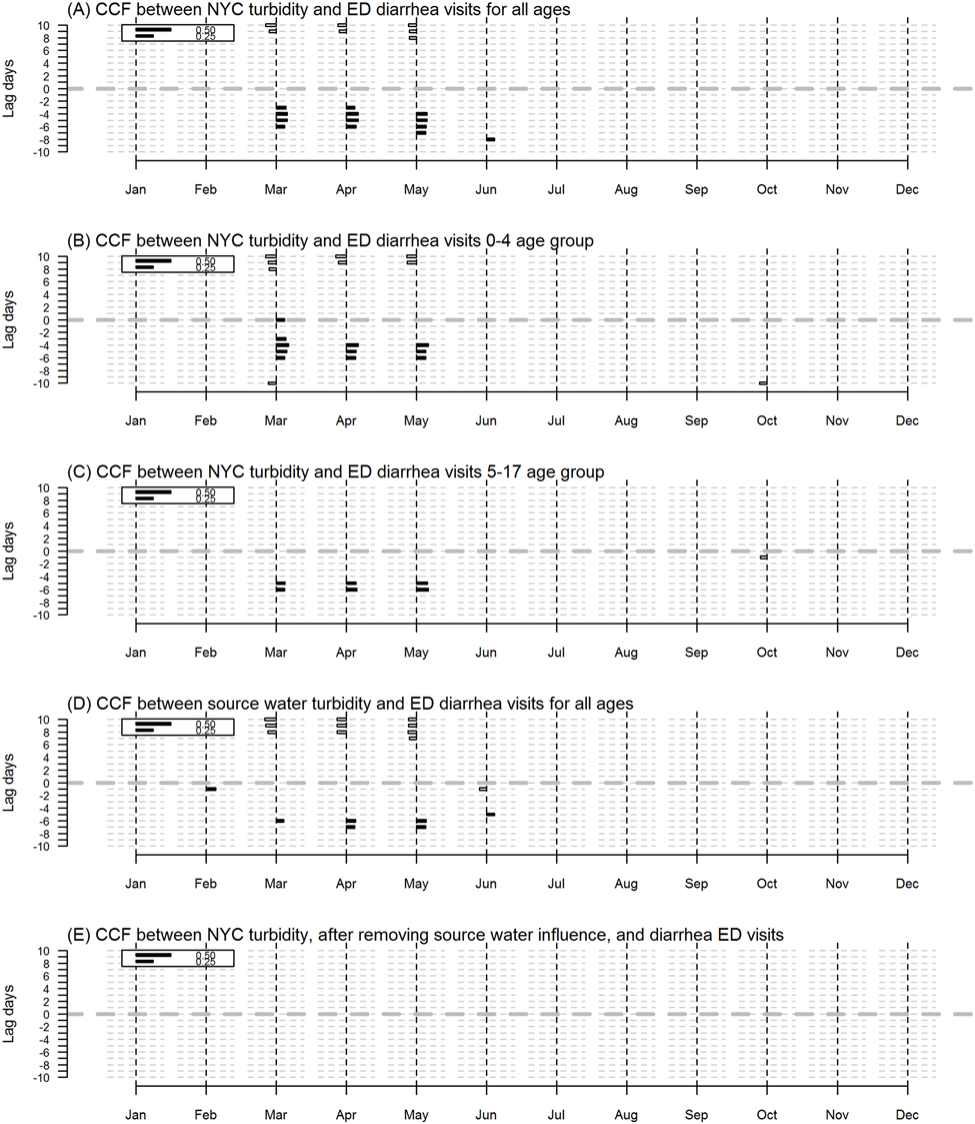
Cross Correlation Function (CCF) results for turbidity and diarrhea ED visits. CCF results for 2002–2009 for A) NYC turbidity and diarrhea ED visits all ages B) NYC turbidity and diarrhea ED visits 0–4 age group C) NYC turbidity and diarrhea ED visits 5–17 age group and D) Source water turbidity and diarrhea ED visits all ages. Each bar shows the degree of correlation between ambient temperature and diarrhea ED visits. Solid bars to the right of the vertical dashed lines indicate positive correlations, hollow bars to the left indicate negative correlations. The bottom half of each panel (below the center line at lag 0), represents where the first variable leads the second and the area above the center line at lag 0 indicates that the second variable leads the first.

The CCF between NYC turbidity and diarrhea ED visits for all ages showed lagged positive correlations between turbidity and diarrhea ED visits ranging from 3–6 days in the spring (Fig 4). A similar CCF pattern was observed in the 0–4 age group and the 5–17 age group but not in the older age groups (18–64 years and 65 years and over) (not shown). NYC turbidity and source water turbidity were highly positively correlated; source water turbidity led NYC turbidity. The CCF between source water turbidity and diarrhea ED visits for all ages showed consecutive lagged positive correlations with turbidity leading diarrhea ED in the months April and May starting at day 6 (Fig 4).

Because much of the temporal variation of NYC turbidity appeared to be explained by source water turbidity, we further examined if the temporal variation unique to NYC turbidity was associated with diarrhea ED visits. We first fit a Gaussian regression model of NYC turbidity on source water turbidity lagged 0, 1, and 2 days in, and then examined the CCF of the model residuals and diarrhea ED visits. Most (83%) of the variance of NYC turbidity was explained by source water turbidity. With the influence of source water turbidity removed, the model residual NYC turbidity showed no association with diarrhea ED visits (Fig 4E). Thus, the association between NYC turbidity and diarrhea ED visits appears to be explained by the influence of source water turbidity. In addition, this analysis was repeated using Catskill and Delaware watershed turbidity data alone (Croton removed) and results showed a similar association in the spring season among the younger age groups (not shown).

**Fig 5:**
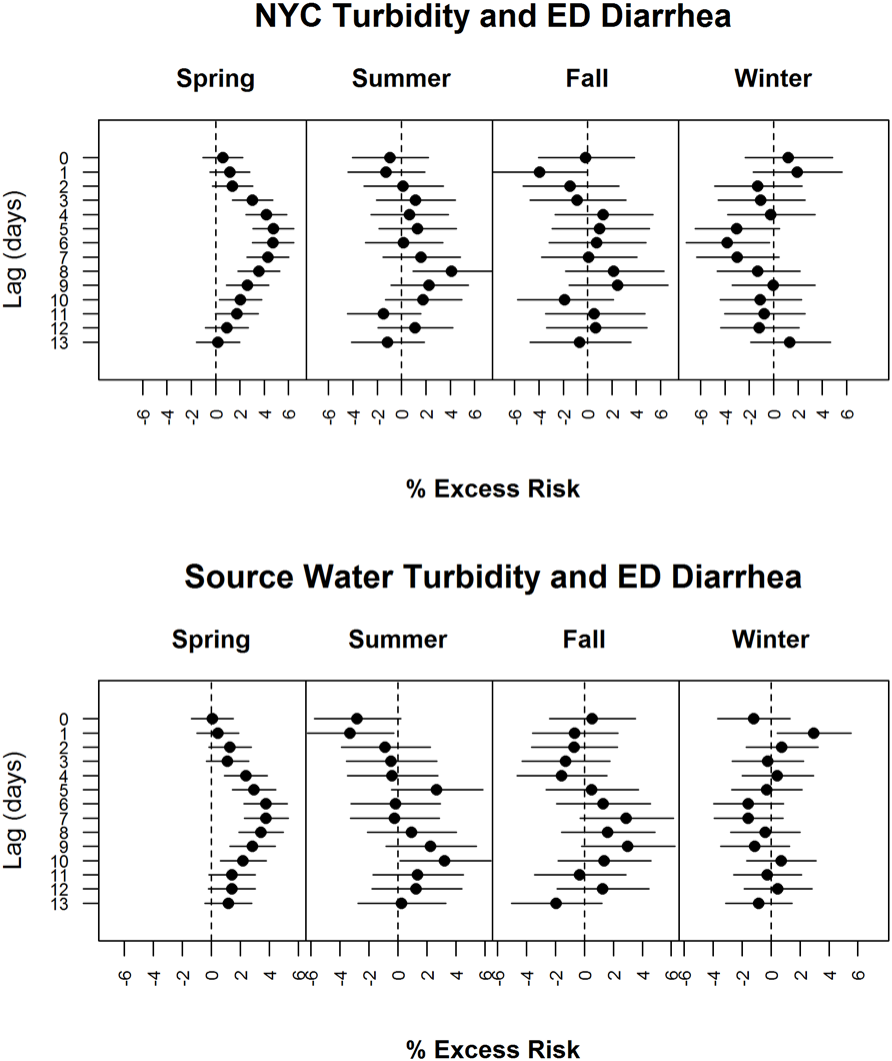
Regression results for turbidity and diarrhea ED visits. Regression results for A) NYC turbidity and diarrhea ED visits for all ages and B) Source water turbidity and diarrhea ED visits (2002–2009). The regression analysis between turbidity and diarrhea ED visits for all ages shows 0% excess risk (ER) at day 0 and an increasing %ER which peaks at 4% at a lag of day 6 in the spring season. There is no consistent increased excess risk in other seasons.

The regression analysis between NYC turbidity and diarrhea ED visits for all ages showed positive associations at consecutive lags, peaking at estimated excess risk of about 5% per inter-quartile-range at a lag of day 6 in the spring season (Fig 5). The 95% confidence bands for the estimates in spring were consistently narrower than those in other seasons mainly due to greater variance in turbidity in spring. There was no consistent association in other seasons. The age-specific model results for the 0–4 and 5–17 year age groups in the spring season were similar to the all age model; no association in older age groups were seen in any season (not shown). The regression analysis between source water turbidity and diarrhea ED visits for all ages exhibited associations similar to those for NYC turbidity and diarrhea ED visits in the spring, except that the estimated excess risk peaks at lag day 7, consistent with the observation that NYC turbidity showed the strongest association with 1-day lagged source water turbidity. There was no consistent increased excess risk in other seasons (Fig 5). With regard to other covariates, temperature on the same day was marginally positively significantly associated with diarrhea ED visits except summer. The average of lag 1 through 3 days’ temperature was a marginally significant negative predictor of diarrhea ED visits in winter only. Diarrhea ED visits were consistently significantly lower on weekends across seasons.

**Fig 6:**
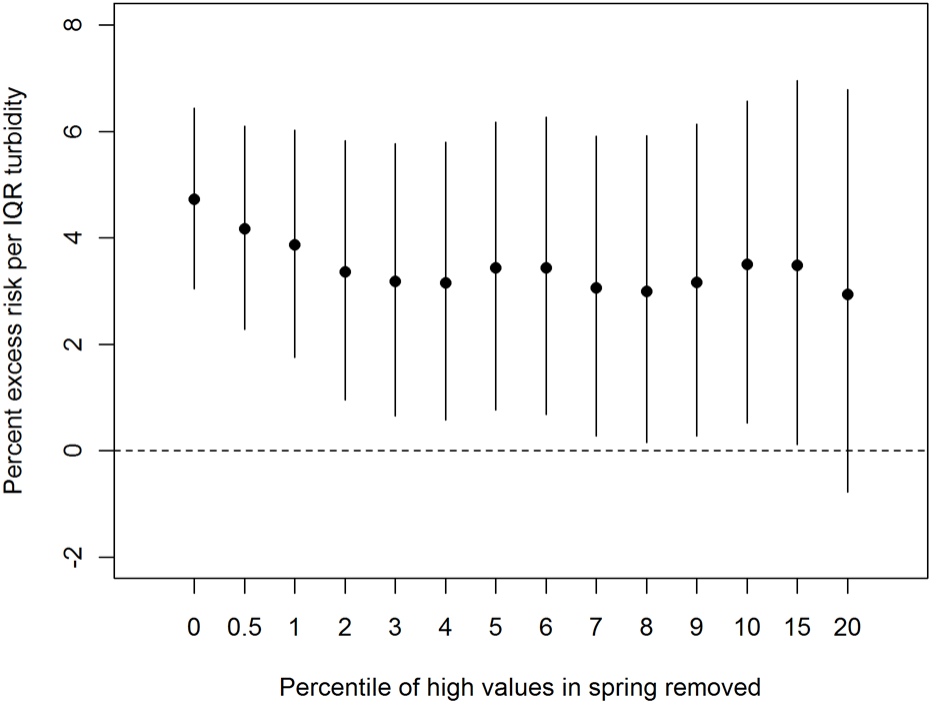
Sensitivity of diarrhea ED risk estimate at lag day 6 to removal of up to the highest 20% of turbidity values, 2002–2009.

The regression diagnostics did not exhibit any condition suggesting a violation of model assumptions. The negative binomial GLM regression model results produced nearly identical point estimates and slightly narrower 95% confidence bands (standard errors for the negative binomial model were <5% smaller) as the main quasi-liklihood Poisson model (results not shown). Thus, there is no practical difference in using a quasi-likelihood Poisson model and a negative binomial model. In a sensitivity analysis (Fig 6), as the highest turbidity values were removed, the excess risk for diarrhea ED visits at lag day 6 in the spring decreased and the confidence intervals widened likely due in part to less variation in turbidity; the estimated risk was not significant when the highest 20^th^ percent of values was removed. Estimated risks were not sensitive to alternative degrees of freedom to adjust for within-season temporal trends or temperature (S1 Fig). Distributed lag models, which took into consideration multi-day associations over 0- to 13-day lags with polynomial forms considering 2^nd^ to 4^th^ degrees (S2 Fig) yielded excess risk estimates for the spring season ranging from 11% to 13% per IQR increase in turbidity, roughly doubling the estimated risk from the single individual lag model, but the associated confidence bands also widened.

The estimated risks were not sensitive to subsets of years used (S3 Fig) or to alternative temperature model specifications (S4 Fig). Overall, seasonal variation unrelated to turbidity dominated the variation of daily diarrhea ED visits. When an annual regression model was fit with seasonal cycles and trends only (i.e., natural splines of study days), 88% of the deviance was explained by these temporal variations. An addition of a day-of-week variable in the model explained an additional 3% of the deviance. Further including turbidity variables with season interactions yielded an additional 0.4% increase in the deviance explained.

## Discussion

Turbidity in the NYC distribution system was positively associated with an increase in diarrhea ED visits in the spring season only, among the youngest age groups, peaking at approximately 6 to 7 day lag. This association accounted for a very small proportion of temporal variation in diarrhea ED visits, with the majority due to seasonal illness patterns unrelated to source water turbidity. Source water turbidity was the major contributor of variation in NYC distribution system turbidity and the primary driver of the association of NYC turbidity with diarrhea ED visits seen in this analysis, not turbidity originating within the distribution system. Source water turbidity primarily reflects turbidity from the Catskill and Delaware systems. There are multiple regulations governing turbidity limits and details regarding turbidity results and regulatory compliance for the study years are available in the NYC DEP Drinking Water Supply and Water Quality Reports [16].

The association was present after adjustment for temporal patterns and weather to account for seasonal illness and changes in care-seeking patterns and was robust to differences in model specifications. The pattern of the multi-day associations, which showed a steady increase, a mode around day 6, and a steady decrease, was consistent with a variable between exposure to an infectious agent and presentation to the ED with complaints of diarrhea that would be expected based on differences among individuals in exposure, susceptibility, and care seeking behaviors. This pattern was inconsistent with a chance finding.

The results also suggest that the highest turbidity values contribute disproportionately to the association between NYC turbidity and diarrhea ED visits. This association was limited to the spring season, when turbidity levels are highest and most variable. Excluding the highest turbidity values in the spring also reduced the strength of associations (Fig 6). It is not clear if the greater variation in turbidity in the spring or the nature of turbidity in the spring is most relevant. While precipitation levels can be high in the spring, they tend to peak in summer or fall, suggesting that other seasonal factors may be relevant to this association such as greater surface runoff from snowmelt and limited tree foliage cover in the spring.

Previous analyses of turbidity and GI illness have shown a range of results. In Mann et al.’s review (2007) they identified six “relevant good quality” studies. They were studies using data from: Philadelphia [2, 4], Milwaukee [12], Greater Vancouver, Canada [5], Edmonton, Canada, [6], and Quebec, Canada [23]. The methods, outcome, and effect measures reported in these studies varied, and therefore, Mann and her colleagues provided a qualitative summary. They concluded that “It is likely that an association between turbidity and GI illness exists in some settings or over a certain range of turbidity.” Since the Mann et al. review, Tinker et al. (2010) conducted an analysis of turbidity and ED visits related to GI illness in Atlanta, GA, which we considered as relevant based on the Mann et al.’s evaluation criteria. Overall, one study [6] found no association between turbidity and GI illness, and six of the seven found variable positive associations- some only found associations with pre-treatment turbidity. Comparing the effect size estimates reported across studies, especially based on per turbidity unit basis, is challenging because the interpretation of turbidity differs for pre- and post- filtration values.

We found a positive association in the younger age groups, consistent with studies of turbidity and GI illness in children specifically, though lag times, and other factors varied between studies [2, 3, 5, 12], and similar to Tinker et al. and the Morris et al. studies, which found the strongest associations across ages in the youngest age groups. We did not find a positive association of turbidity and GI illness in older adults, in contrast to some previous studies [4, 5, 12].

While most of these previous studies controlled for seasonality, none conducted separate analyses by season as in our study. The amount and source of turbidity can vary by season in a watershed and seasonal analysis helped produce a more focused result using a general indicator. Regional variation in seasonal influences on turbidity may make this approach useful in additional locations. Some recent studies have examined the relationship between GI illness and seasonal hydrological factors, rainfall and stream flow, and found positive associations [24, 25]. While Drayna et al. analysis found that GI illness increased after 4 days, Jagai et al. found that GI illness peaks preceded stream flow peaks. Thus, further research is needed to understand the nature of the association with different hydrological parameters as some may be indirectly associated.

NYC has historically been an unfiltered drinking water system, with water supplied from the extensively protected Catskill, Delaware, and Croton watersheds. Associations between turbidity and endemic GI illness reported in past studies were found in both filtered [2–4] and unfiltered [5] drinking water systems. Lim et al. and Tinker et al. examined both raw water and post-treatment water turbidity. Tinker et al. found an association with raw water only. Lim found no association with raw water or post-treatment water turbidity.

There are several limitations in this study. Given available data, this study used an ecological study design. It is difficult to characterize the nature of association in terms of individual risk since we did not have individual-level exposures and individual outcomes. The association was for the daily variation of turbidity as observed in the distribution system with the citywide variation of ED visits for GI illness. The source water data, which was analyzed as a supplement to the NYC water quality data, had several limitations. Water quality data from the source water reservoir systems was limited to 3 sites—one from each reservoir system and this data varied in completeness. Flow data available for this study was measured at a point in the system after Catskill and Delaware water had already mixed so these reservoir systems were not assessed independently. There was 19.8% missing data for the Croton system, however the overall contribution of the Croton system to the total flow was relatively small so it did not have much impact on the flow-weighted average turbidity for source water.

Data necessary to conduct a study on a smaller geographic scale within NYC was not available, and therefore, this study could not address the relationship of localized turbidity increases with local GI illness. Other water quality data that were available to us did not meet the requirements for this type of analysis. For example, microbial indicator results reported were predominantly below the detection limit. Thus, we could not examine this as an alternative indicator of water quality in this study.

Turbidity as an indicator of water quality is also limited. A basic turbidity measurement alone does not provide information about the type or source of suspended particles. Regional differences in watersheds can influence the source and composition of particles contributing to turbidity and this may explain differences across some studies. For example, Lim et al. (2002) suggest that the drinking water source for Edmonton, Canada is strongly influenced by clay and silt particles associated with glacial melt and runoff, which may help explain the lack of association of GI illness despite relatively high turbidity values.

ED visits for GI illness are likely an underestimate of overall community GI illness, as most people with GI symptoms do not seek medical care or seek care in outpatient settings. However, syndromic surveillance data are relatively quickly and easily measured and the speed of electronic data availability make diarrhea ED visits a potentially useful and timely albeit non-specific indicator of GI illness to help assess whether a potential water contamination event is having a population level impact on GI illness. Laboratory confirmation of waterborne pathogens is more specific, but it can take days or weeks to receive these results [11].

While these results should be viewed with consideration of any study limitations discussed above, the strength of this work and the selected model are reflected in the ability to detect the small magnitude of an association. Multiple sensitivity analyses support the robustness of the association. These findings interpreted along with findings from other published water quality and health studies, support the need for additional research in this area to elucidate the cause of the association and to identify an indicator or metric that can be more readily compared across regions. Given that turbidity is an indicator only and the causative agent is unknown, further investigation of turbidity levels in the spring season is needed, but no specific turbidity alert level is recommended at this time.

Many types of data are used to monitor the NYC drinking water system as part of ongoing regular surveillance such as physical, chemical, and biological measures of water quality as well as public health surveillance data including syndromic data. This study provided a first step towards more complete understanding of the relationship between water quality and syndromic surveillance data in NYC and results will be considered in the context of the overall water quality monitoring system. No single indicator alone will be used to make management decisions, rather supporting information will be used to provide context to understand alerts from various systems.

In 2013, NYC started providing enhanced UV disinfection of its Catskill/Delaware water supply, and soon will be able to provide enhanced filtration and disinfection of its Croton water supply. It may be useful to repeat this analysis when sufficient data are available to assess whether the association between turbidity and GI illness can still be observed.

## Conclusions

This study identified a small association between turbidity and diarrheal illness in NYC. A large proportion of the strong seasonal variations in diarrhea ED visits likely reflect many different causes of diarrheal illnesses. Smaller scale geographic analysis may provide further information on associations between turbidity and GI illness. Integrated analysis of turbidity data and syndromic surveillance data, as part of overall drinking water surveillance, may be useful for enhanced situational awareness of possible risk factors that can contribute to GI illness. Elucidating the causes of turbidity-GI illness associations including seasonal and regional variations would be necessary to further inform surveillance needs.

## Supporting Information

S1 Fig Examination of risk estimates as a function of alternative degrees of freedom (df) for seasonal adjustment. (PDF)

S2 Fig Fitted polynomial distributed lag models. Fitted polynomial distributed lag models for lag 0 through 13 day turbidity and all-age diarrhea ED visits.
(PDF)

S3 Fig Sensitivity analysis of excess risk of diarrhea ED visits in spring by time period. Sensitivity of percent excess risk of diarrhea ED visits for all-age group at lag 6 day in a 4th-order polynomial distributed lag model in spring periods using consecutive four years of spring periods.
(PDF)

S4 Fig Sensitivity of percent excess risk of diarrhea ED visits to alternative temperature specifications. Sensitivity of percent excess risk of diarrhea ED visits for all-age group at lag 6 day in a 4th-order polynomial distributed lag model in spring periods using alternative temperature specifications (1) no adjustment for temperature; (2) natural spline of same-day temperature with 3 degrees of freedom and natural splines of the average of lag 1 through 3 days with 3 degrees of freedom (the base model); (3) natural splines of same-day temperature with 3 degrees of freedom and natural splines of the average of lag 3 through 8 days with 3 degrees of freedom; and (4) 4^th^-degree distributed lag model of temperature for lags 0 through 13 days.
(PDF)

S1 Table Summary statistics of NYC daily turbidity data, 2002–2009. (PDF)
