# Supplementary material for: Drinking Water Turbidity and Emergency Department Visits for Gastrointestinal Illness in New York City, 2002-2009
Source: PLoS One. 2015 Apr 28;10(4):e0125071. doi: 10.1371/journal.pone.0125071 (PMC4412479; doi:10.1371/journal.pone.0125071)
Supplement: S1 Fig — (PDF) [file pone.0125071.s001.pdf]

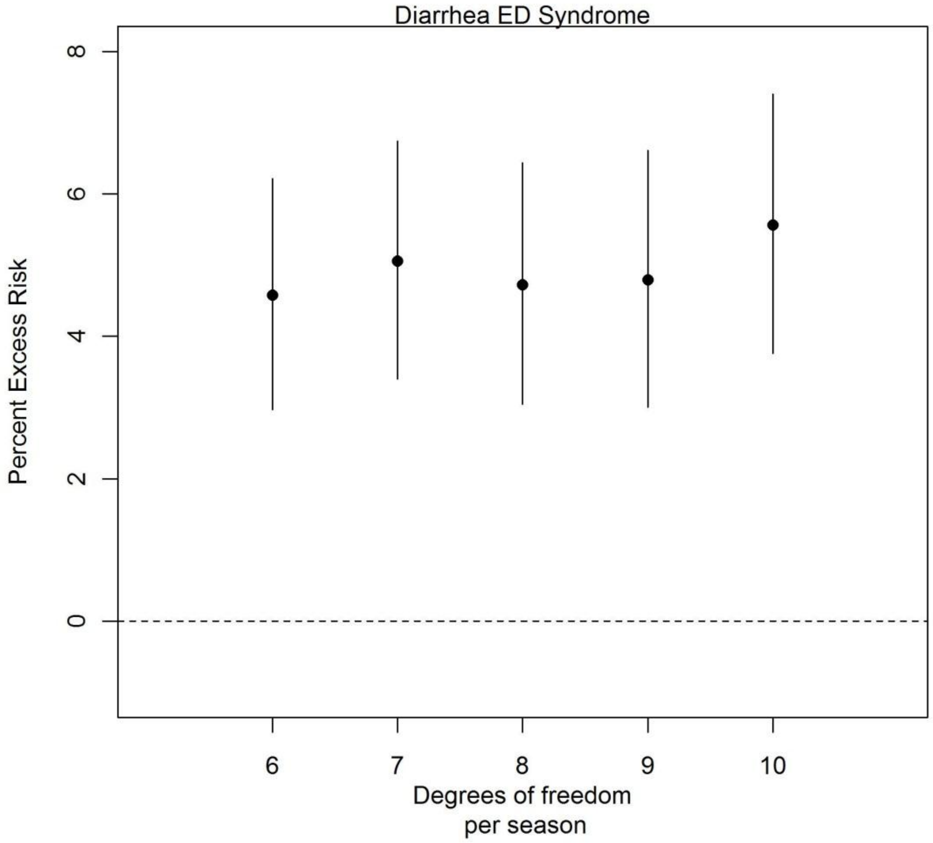

S1 Fig. Sensitivity analysis- examination of risk estimates as a function of alternative degrees of freedom (df) for seasonal adjustment.
