# Supplementary material for: Drinking Water Turbidity and Emergency Department Visits for Gastrointestinal Illness in New York City, 2002-2009
Source: PLoS One. 2015 Apr 28;10(4):e0125071. doi: 10.1371/journal.pone.0125071 (PMC4412479; doi:10.1371/journal.pone.0125071)
Supplement: S2 Fig — Fitted polynomial distributed lag models for lag 0 through 13 day turbidity and all-age diarrhea ED visits. (PDF) [file pone.0125071.s002.pdf]

(i) Second-degree polynomial distributed lag model

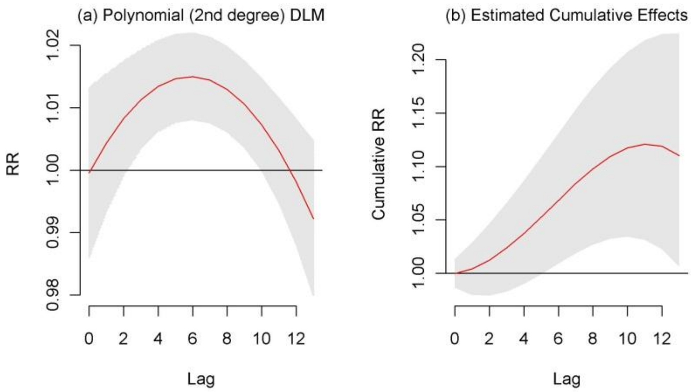

(ii) Third-degree polynomial distributed lag model

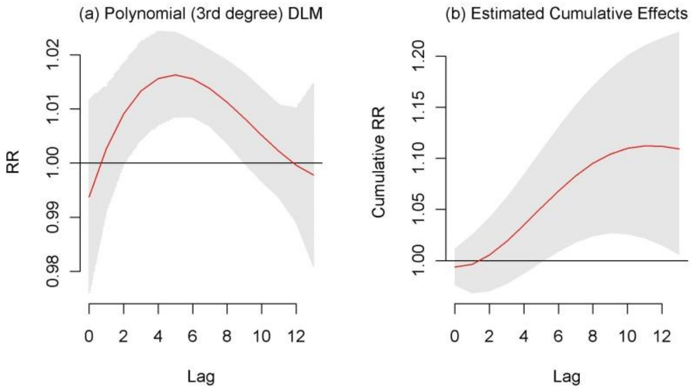

(iii) Fourth-degree polynomial distributed lag model

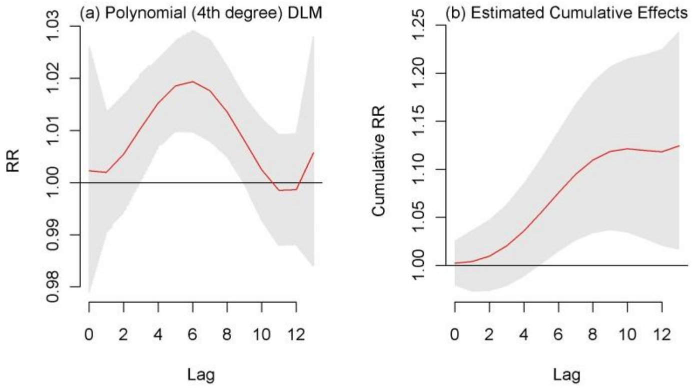

S2 Fig. Fitted polynomial distributed lag models for lag 0 through 13 day turbidity and all-age diarrhea ED visits.
