# Supplementary material for: Drinking Water Turbidity and Emergency Department Visits for Gastrointestinal Illness in New York City, 2002-2009
Source: PLoS One. 2015 Apr 28;10(4):e0125071. doi: 10.1371/journal.pone.0125071 (PMC4412479; doi:10.1371/journal.pone.0125071)
Supplement: S3 Fig — Sensitivity of percent excess risk of diarrhea ED visits for all-age group at lag 6 day in a 4th-order polynomial distributed lag model in spring periods using consecutive four years of spring periods. (PDF) [file pone.0125071.s003.pdf]

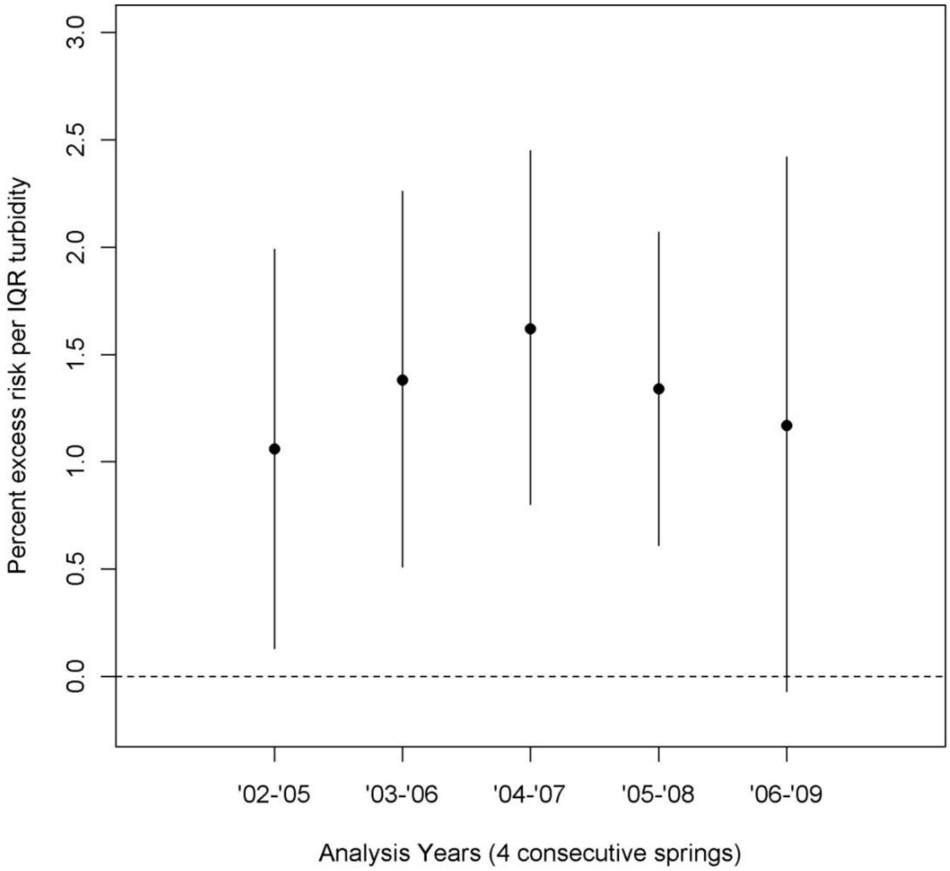

S3 Fig. Sensitivity analysis of excess risk of diarrhea ED visits in spring by time period. Sensitivity of percent excess risk of diarrhea ED visits for all-age group at lag 6 day in a 4th-order polynomial distributed lag model in spring periods using consecutive four years of spring periods.
