# Supplementary material for: Drinking Water Turbidity and Emergency Department Visits for Gastrointestinal Illness in New York City, 2002-2009
Source: PLoS One. 2015 Apr 28;10(4):e0125071. doi: 10.1371/journal.pone.0125071 (PMC4412479; doi:10.1371/journal.pone.0125071)
Supplement: S4 Fig — Sensitivity of percent excess risk of diarrhea ED visits for all-age group at lag 6 day in a 4th-order polynomial distributed lag model in spring periods using alternative temperature specifications (1) no adjustment for temperature; (2) natural spline of same-day temperature with 3 degrees of freedom and natural splines of the average of lag 1 through 3 days with 3 degrees of freedom (the base model); (3) natural splines of same-day temperature with 3 degrees of freedom and natural splines of the average of lag 3 through 8 days with 3 degrees of freedom; and (4) 4th-degree distributed lag model of temperature for lags 0 through 13 days. (PDF) [file pone.0125071.s004.pdf]

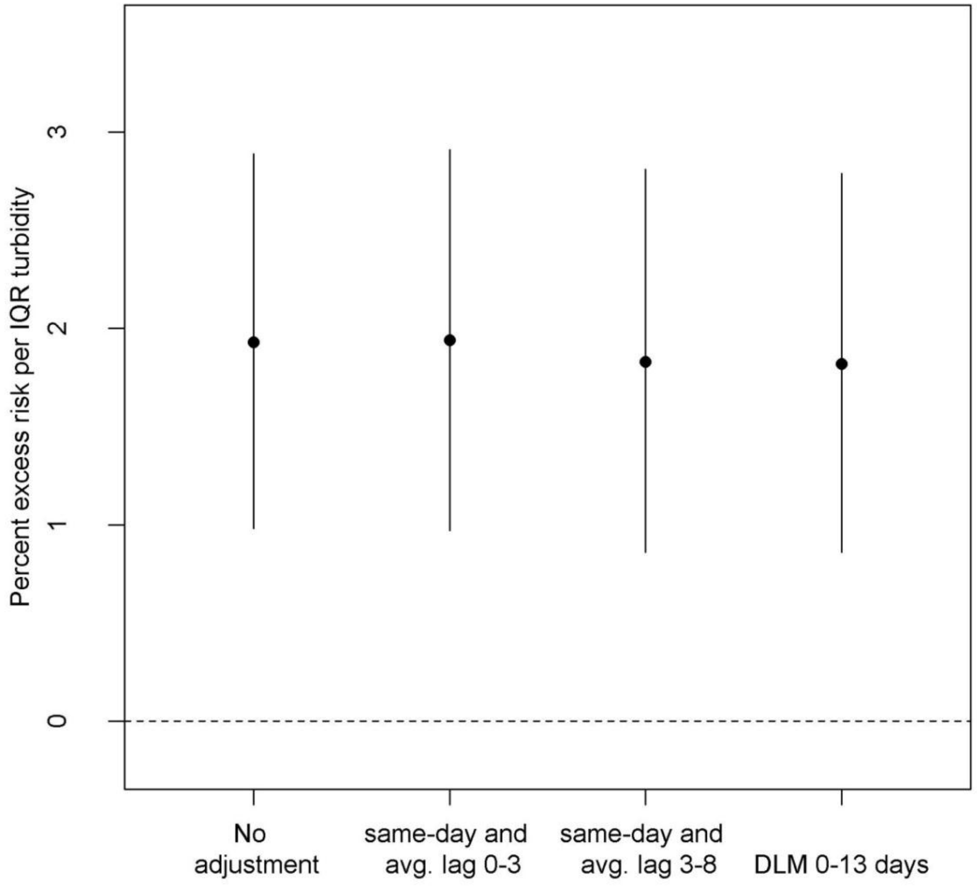

S4 Fig. Sensitivity of percent excess risk of diarrhea ED visits to alternative temperature specifications. Sensitivity of percent excess risk of diarrhea ED visits for all-age group at lag 6 day in a 4th-order polynomial distributed lag model in spring periods using alternative temperature specifications (1) no adjustment for temperature; (2) natural spline of same-day temperature with 3 degrees of freedom and natural splines of the average of lag 1 through 3 days with 3 degrees of freedom (the base model); (3) natural splines of same-day temperature with 3 degrees of freedom and natural splines of the average of lag 3 through 8 days with 3 degrees of freedom; and (4) 4th-degree distributed lag model of temperature for lags 0 through 13 days.
