# Supplementary material for: Drinking Water Turbidity and Emergency Department Visits for Gastrointestinal Illness in New York City, 2002-2009
Source: PLoS One. 2015 Apr 28;10(4):e0125071. doi: 10.1371/journal.pone.0125071 (PMC4412479; doi:10.1371/journal.pone.0125071)
Supplement: S1 Table — (PDF) [file pone.0125071.s005.pdf]

S1 Table: Summary statistics of daily turbidity data

	Years	N sample sites	Mean (ntu)	Median (ntu)	Range (ntu)	N days system running	N days missing data	Percent of source water flow
NYC distribution system turbidity (daily median of available samples from multiple sites)	2002-2009	375	0.97	0.92	0.54-2.38	2922	0	NA
Overall source water turbidity[1] (daily flow-weighted average)	2002-2009	3	0.98	0.95	0.50-2.85	2922	0	100%
Catskill/Delaware turbidity[1,2] (daily average)	2002-2009	2	0.98	0.95	0.50-2.85	2922	26/47	96%
Croton turbidity	2002-2009	1	1.00	1.00	0.50-2.80	1166	578	4%

1- Single outlier (4.6 ntu) excluded from analysis

2- Catskill and Delaware flow data available only as combined flow, contributions by volume from each system equal per NYC DEP
